# Protocol for modeling the repair of intestinal damage by co-culturing mesenchymal stromal/stem cells and intestinal organoids

**DOI:** 10.1016/j.xpro.2025.104292

**Published:** 2025-12-24

**Authors:** Kari-Pekka Skarp, Bahar Yetkin-Arik, Suze A. Jansen, Caroline A. Lindemans, Magdalena J. Lorenowicz

**Affiliations:** 1 Biomedical Primate Research Centre, 2288 GJ Rijswijk, the Netherlands; 2 Center for Molecular Medicine, University Medical Center Utrecht, Universiteitsweg 100, 3584 CG Utrecht, the Netherlands; 3 Regenerative Medicine Center, Uppsalalaan 8, 3584 CT Utrecht, the Netherlands; 4 Centre for Living Technologies, Alliance TU/e, WUR, UU, UMC Utrecht, Princetonlaan 6, 3584 CB Utrecht, the Netherlands; 5 Division of Pediatrics, University Medical Center Utrecht, Utrecht, the Netherlands; 6 Pediatric Stem Cell Transplantation, Princess Máxima Center for Pediatric Oncology, Utrecht, the Netherlands

**Keywords:** Cell biology, Single cell, Cell-based assays, Molecular biology, Signal transduction, Stem cells, Organoids

## Abstract

Mesenchymal stem/stromal cells (MSCs) are known for their regenerative properties. This protocol describes a co-culture system for investigating molecular interactions between MSCs and intestinal epithelial organoids following injury. We outline steps for assessing the immediate effects of MSCs on organoid growth and survival, as well as a model for evaluating longer term responses. The workflow is adaptable and can be readily modified to examine MSC interactions with additional cell types or in different injury contexts.

For complete information on the use and execution of this protocol, please refer to Yetkin-Arik *et al.*

## Highlights

• Procedures for the co-culture of human MSCs and human duodenal epithelial organoids
• Steps for light microscopy-based readout of MSC effects on organoids exposed to injury
• Detailed steps for data quantification using OrganoSeg and OrganoAna tools
• System compatible with downstream analyses using standard cell biology methods

## Abstract (editor highlights)

Publisher’s note: Undertaking any experimental protocol requires adherence to local institutional guidelines for laboratory safety and ethics.

## Graphical abstract

**Figure:**
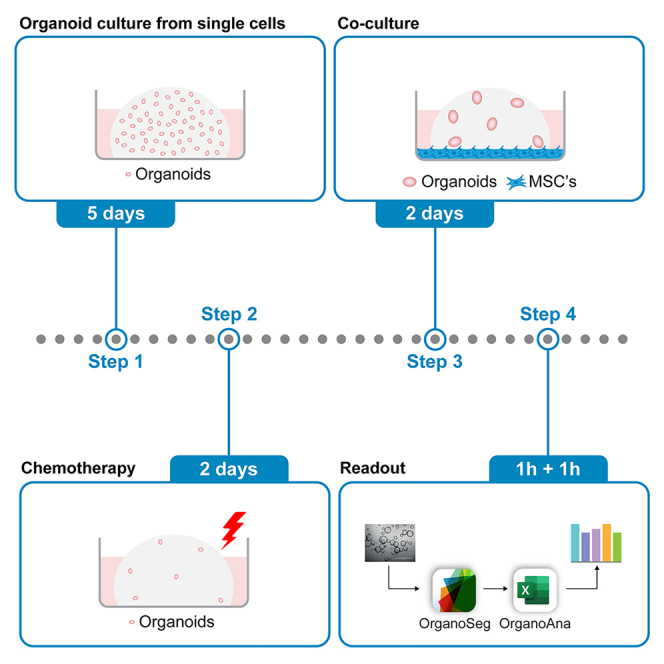

## Before you begin

Allogeneic^1^ MSC infusion is a promising therapy for acute steroid refractory graft-versus-host-disease (aGvHD).^2^^,^^3^ While previous studies have primarily focused on the immunomodulatory capacities of MSCs, very little is known regarding their regenerative properties in relation to organs, such as the intestine. The intestine is the organ most affected by chemotherapy-induced damage, which is a prerequisite to GvHD development, and in the progression of GvHD itself.^4^ Our newly developed model allows to study the interplay between MSCs and duodenal epithelium in an environment that mimics more closely the *in vivo* situation of chemotherapy conditioning than currently available *in vitro* systems and at the same time offers a lower complexity than animal models.

In short, duodenal organoids are seeded in matrigel and treated with busulfan, a DNA alkylating agent and a known cytotoxin. Busulfan is removed and MSCs are introduced to the culture. After several days, the wells are imaged with a light microscope and the size and number of organoids is quantitated. Due to the number of organoids in one droplet, this assay is sensitive and has substantial statistical power and can be scaled up. This protocol uses an established human duodenal organoid line, which has been passaged over ten times. The generation of organoids from biopsies has been described elsewhere.^5^

The visual analysis of organoids may require advanced imaging and analysis tools, which takes time and investment and may be tedious to use. Our goal here is to present a complete workflow from cells to numbers to figures using easy to use free tools and a standard cell culture light microscope enabling low threshold of adoption for anyone interested.

All steps in this protocol should be performed in a laminar flow hood to prevent contamination. Importantly, cells should always be kept on ice during handling unless stated otherwise. It is also recommended to confirm that the cells used are mycoplasma negative. All cell culture steps in this protocol are carried out at 37°C in a humidified incubator with 5% CO_2_.

### Innovation

We developed a 3D co-culture model of human small intestinal organoids and bone marrow-derived mesenchymal stromal/stem cells to study epithelial injury and repair processes relevant to chemotherapy-induced damage. Rather than aiming to reproduce full intestinal organogenesis, this system provides a controllable and reproducible environment to investigate epithelial-stromal crosstalk under defined injury conditions, offering a complementary approach to existing iPSC-derived organoid models that recapitulate more complex tissue organization.

A key strength of this platform is its ability to directly assess the regenerative influence of stromal signaling on damaged intestinal epithelium, providing mechanistic insight into pathways with therapeutic relevance for conditions such as acute steroid-refractory GvHD. Importantly, our model also reflects the clinical setting, where bone marrow-derived MSCs are used therapeutically in patients.

Beyond this context, the system is highly adaptable and can be extended to other injury paradigms, such as irradiation, or to explore stromal-epithelial interactions within the tumour microenvironment. Future iterations of the model will allow incorporation of primary intestinal mesenchymal subtypes to study epithelial-stromal interactions in a more physiologically relevant context. Its scalability and compatibility with high-throughput analysis enable comparative studies across donors or patient-derived organoids, supporting applications in precision and regenerative medicine.

Finally, our freely accessible OrganoAna tool automates the analysis of OrganoSeg-derived imaging data, including integrated statistical evaluation, thereby facilitating efficient and reproducible interpretation. Together, the co-culture platform and automated analysis pipeline provide informative framework for dissecting MSC-epithelium communication and refining therapeutic strategies in intestinal regeneration and disease.

### Institutional permissions

Institutional permission for obtaining and studying clinical samples is needed as well as informed consent from each individual patient or healthy donor. For the results described in this protocol*,* human healthy duodenal epithelial organoids were cultured from banked frozen organoids that had been previously generated from biopsies obtained during duodenoscopy of healthy human controls.^6^^,^^7^ All healthy controls had been investigated for celiac disease but turned out to have no pathology. They had previously provided written informed consent to participate in this study according to a protocol reviewed and approved by the review board of the UMC Utrecht, the Netherlands (protocol STEM study, METC 10-402/K). MSCs were isolated from third-party non-HLA-matched healthy bone marrow donors as approved by the Dutch Central Committee on Research Involving Human Subjects (CCMO, Biobanking bone marrow for MSC expansion, NL41015.041.12) and all samples were obtained with written informed consent from the bone marrow donor or parent/legal guardian of the donor.

### Preparation of Wnt-3A- and Noggin-conditioned media

**Timing: ∼3 weeks**

1. Carefully study Vonk et al.^5^ and prepare Wnt-3A and Noggin conditioned media as described therein (WCM and NCM, respectively).

***Note:*** Both Noggin and Wnt-3A are available also commercially. Noggin can be purchased either as a powder or frozen NCM while Wnt-3A is sold as a powder (for example from ImmunoPrecise or SinoBiological). However, purchasing these reagents increases considerably the costs of experiments.

### Production of R-Spondin I-conditioned medium

**Timing: ∼2.5 weeks**

***Note:*** R-Spondin I is also commercially available as a powder or frozen RCM (for example from ImmunoPrecise or SinoBiological).

2. Thawing and growing the R-Spondin I producing HEK293T cell line.
  a. Prepare DMEM +/+ and AdDF+++.
  b. Thaw a vial of HEK293T-HA-Rspo1-Fc cells in DMEM +/+ with 300 μg/ml Zeocin into a T-75 flask.
  c. Upon confluency, split the T-75 into 2 x T-175 flasks with 25 ml of DMEM +/+ and Zeocin.
  d. Upon confluency, split the 2 x T-175 flasks into 12 x T-175 flasks with 25 ml of DMEM +/+. Add Zeocin only into 2 bottles.
  e. When the 10 flasks without Zeocin are confluent, remove DMEM +/+ and add 50 ml AdDF+++ per bottle and incubate one week.
  f. Collect medium into 50 ml tubes, spin down for 5 min at 650 g to remove cell debris and filter through a 0.22 μm pore size filter.
  g. Aliquot in usable volumes (such as 20 ml) and store at −20°C. These stocks can be used for one year.
    **CRITICAL:** Validate the produced RCM by comparing the growth of organoids in it to commercial R-Spondin I or previous batches that were known to work. RCM should not undergo additional freeze-thaw cycles.
  h. Use the remaining 2 bottles (with Zeocin) to start another culture. The cells can be used for 10–12 passages consecutively for RCM production.

### Preparation of mesenchymal stem/stromal cell growth medium

**Timing: ∼30 min**

3. Prepare reagents for the MSC growth medium.
  a. Dissolve 50 μg of bFGF in 5 ml PBS and aliquot into 10 x 500 μl tubes (10 μg/ml).
  b. Store at −20°C for up to a year, avoid repeated freeze/thaw cycles.
  c. Dissolve 0.579 g of L-ascorbic acid phosphate in 40 ml of demi-water (=50 mM).
  d. Prepare the medium and sterile filter through a 0.22/0.45 μm membrane.

## Key resources table

**Table:** 

REAGENT or RESOURCE	SOURCE	IDENTIFIER
**Chemicals, peptides, and recombinant proteins**		

Advanced Dulbecco’s Modified Eagles Medium with Nutrient Mixture F-12 Hams (Ad-DF), 500 ml	Thermo Fisher Scientific	12634
DMEM, high glucose, GlutaMAX Supplement, pyruvate	Thermo Fisher Scientific	31966–021
α-MEM	Thermo Fisher Scientific	22571–020
Fetal bovine serum (FBS)	Biowest	S1810
Penicillin/Streptomycin	Thermo Fisher Scientific	15140–122
GlutaMAX	Thermo Fisher Scientific	35050
HEPES (1 M)	Thermo Fisher Scientific	15630–056
Phosphate Buffered Saline	Thermo Fisher Scientific	10010056
Phosphate Buffered Saline 0 (without Ca^2+^ and Mg^2+^)	Thermo Fisher Scientific	14190250
L-glutamine	Thermo Fisher Scientific	25030–024
bFGF	Invitrogen	PHG0026
L-ascorbic acid phosphate	Sigma-Aldrich	A8960-5gr
TrypLE Express	Thermo Fisher Scientific	12604013
Dimethyl sulfoxide (DMSO)	Sigma-Aldrich	276855-1L
B27 supplement	Thermo Fisher Scientific	17504–044
N-Acetylcysteine	Sigma-Aldrich	A9165
Nicotinamide	Sigma-Aldrich	N0636
SB202190 (p38 MAPK inhibitor)	Sigma-Aldrich	S7067
A83-01 (TGFb type I Receptor inhibitor)	Tocris	2939
mEGF	PeproTech	AF-100-15
Matrigel	Corning	356231
Y-27632 Dihydrochloride (ROCK inhibitor)	Abmole bioscience	Y-27632
G418 (100 mg/mL)	InvivoGen	ant-gn-5
Zeocin (100 mg/mL)	Thermo Fisher Scientific	R25001
Gentamicin	Thermo Fisher Scientific	15710–049
Vancomycin	Sigma-Aldrich	861987- 250mg
Busulfan	College ter beoordeling van geneesmiddelen	N/A

**Experimental models: Cell lines**		

HEK293 – Noggin-Fc producing cell line	Obtained from Prof. Hans Clevers laboratory	
HEK293T-HA-Rspo1-Fc producing cell line	Obtained from Prof. Calvin Kuo laboratory	Ootani et al., 2009
L-Wnt3A producing cell line	Obtained from Prof. Hans Clevers laboratory	
Human duodenal epithelial organoids	UMCU Biobank	
Primary human mesenchymal stromal/stem cells	Cell Therapy Facility of UMCU	

**Software and algorithms**		

OrganoSeg	Borten et al., 2018	https://github.com/JanesLab/OrganoSeg
OrganoAna	This manuscript DOI https://doi.org/10.5281/zenodo.17602501	https://github.com/B-P-R-C/Lorenowicz/blob/main/OrganoAna%20-%20ExpectedResults.xlsm
Microsoft Excel	Microsoft Corporation	https://office.microsoft.com/excel

**Other**		

Serological pipettes	No recommended vendor	N/A
Micropipette set (10–1,000 μL)	No recommended vendor	N/A
Micropipette filter tips (10–1,000 μL)	No recommended vendor	N/A
Micropipette tips without filters (10 μL for breaking up organoids, 200 μL for aspirating media from wells)	No recommended vendor	N/A
15 mL conical tubes	No recommended vendor	N/A
50 mL conical tubes	No recommended vendor	N/A
1.5 mL microcentrifuge tubes	No recommended vendor	N/A
T-75 cell culture flasks	No recommended vendor	N/A
T-175 cell culture flasks	No recommended vendor	N/A
145 mm petri dishes	No recommended vendor	N/A
24-well plates	No recommended vendor	N/A
48-well plates	No recommended vendor	N/A
0.22/0.45 μm filters for cell culture media	No recommended vendor	N/A
0.22 μm filters for syringes	No recommended vendor	N/A
Light microscope with 1.25x-4x objective for acquiring images	No recommended vendor	N/A

## Materials and equipment

**Table:** DMEM +/+

Reagent	Final conc.	Volume
DMEM	89 %	445 ml
FBS	10 %	50 ml
P/S	1 %	5 ml
**Total**	**N/A**	**500 ml**

Store at +4°C for up to 1 month.

**Table:** AdDF+++

Reagent	Final conc.	Volume
AdDF	97 %	485 ml
HEPES	1 %	5 ml
GlutaMAX	1 %	5 ml
P/S	1 %	5 ml
**Total**	**N/A**	**500 ml**

Store at +4°C for up to 2–3 months.

**Table:** MSC growth medium

Reagent	Final conc.	Volume
α-MEM	87.6 %	438 ml
FBS	10 %	50 ml
P/S	1 %	5 ml
L-glutamine	1 %	5 ml
bFGF	1 ng/ml	50 μl
L-ascorbic acid phosphate	200 μM	2 ml
**Total**	**N/A**	**500.05 ml**

Store at +4°C for up to 3 weeks.

**Table:** EM −/−/−

Reagent	Final conc.	Volume
AdDF+++	16.7 %	16.7 ml
B27	2 %	2 ml
N-acetylcysteine	1.25 mM	250 μl
Nicotinamide	10 mM	1 ml
A83-01	500 nM	10 μl
SB202190	10 μM	33 μl
mEGF	50 ng/ml	10 μl
**Total**	**N/A**	**20.003 ml**

Store at +4°C for up to 10 days. The preparation and storage of individual reagents has been described in Vonk et al.^5^

**Table:** EM +/+/+

Reagent	Final conc.	Volume
EM −/−/−	N/A	20 ml
NCM	10%	10 ml
RCM	20%	20 ml
WCM	50%	50 ml
**Total**	**N/A**	100 ml

Store at +4°C for up to 10 days.

## Step-by-step method details

### Handling and passaging of human duodenal epithelial organoids

**Timing: ∼2 h**

This section describes the culture of human duodenal organoids.

**CRITICAL:** Prewarm 24/48-well plates to 37°C by placing them in the incubator on the previous day. This will ensure the matrigel droplets maintain their form and area and do not spread on the plate. If prewarmed plates are not available, place a fresh plate into 37°C for at least 3 h before commencing cell culture. Prewarming for 16 h facilitates the workflow, but it is not critical.

***Note:*** Handling and passaging of human colon organoids have been well described in great detail by Vonk et al.^5^ Due to the similarity of the organs, we cultivate human duodenal organoids using the same protocol with a couple of technical differences described below, which facilitate the mechanical breakdown of organoids.

1. Preparations.
  a. Prepare cold AdDF+++ and warm (37°C) EM +/+/+.
  b. Keep matrigel diluted 50 % with EM +/+/+ on ice.
2. Passaging of human duodenal epithelial organoids.
  a. Aspirate the medium from the well(s).
  b. With a 1000 μl tip, pipette 1 ml cold AdDF+++ into the well to flush the organoids out of the matrigel. Use the pipette tip gently to collect remaining matrigel pieces. Up to 12 wells may be collected in this manner.
  c. Transfer the organoids into a 1.5 ml eppendorf tube, keep on ice all the time.
  d. Connect a p10 tip (without a filter) to a p1000 tip and pipette vigorously up and down 10–15 times.
    ***Note:*** This is most efficient when the tip is pushed to the bottom of the tube almost perpendicularly, thus leaving an even smaller opening for the organoids to pass through during up and down pipetting, shearing them in the process. Use the full volume of the pipette but try to avoid excessive foaming. Use a microscope to monitor the process and continue until the organoids are small enough (Figure 1).
    **Figure 1:**
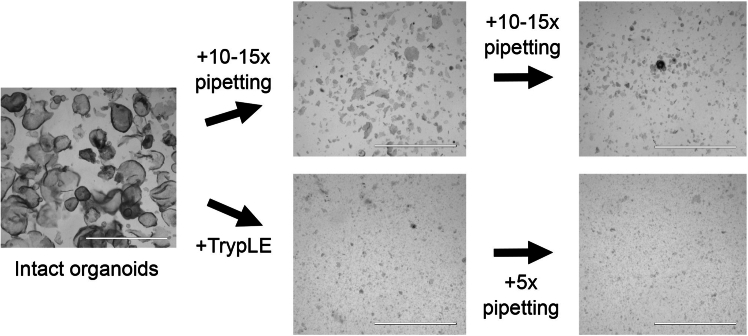
Example images of the process of breaking down organoids Collected organoids can be broken down either mechanically through up/down pipetting, or first enzymatically and then by pipetting up/down. The first is sufficient for weekly organoid culture maintenance while in the latter method the organoids are dissociated into single cells. Scale bar is 2000 μm.
  e. Transfer the cells into a 15 ml tube with 12 ml cold AdDF+++ and centrifuge at 200 g for 5 min at +4°C.
  f. Aspirate the medium and resuspend the organoid pellet in 100–200 μl of 50 % matrigel, depending on how many wells were collected.
  g. Make test droplets, check density under a microscope and dilute with additional matrigel if necessary. As suggested by Vonk et al.^5^ the density should be 15–30 structures per droplet.
  h. Allow the droplets to solidify at 37°C for 15 minutes and then carefully add 500 μl warm EM +/+/+ into the wells.
  i. Place the plate into a 37°C incubator for 2 days. Change media every 2–3 days (for example Mon-Wed-Fri).

### Handling and passaging of mesenchymal stromal/stem cells

**Timing: ∼1 h**

These steps explain the passaging, thawing, and freezing of MSCs

**CRITICAL:** MSCs may exhibit different growth rates, colony-forming capabilities or differentiation properties depending on the donor as well as FBS used in the culture medium. It is therefore important to test that the FBS used is compatible with the MSCs.

3. Preparations.
  a. For freezing, prepare an appropriate amount of 2x MSC freezing media by combining DMSO and MSC growth media in 1:4 ratio (20 % DMSO). In this protocol we need 3 ml of 2x MSC freezing media as we process one confluent T-175 flask.
  b. Prepare and prewarm MSC growth medium and TrypLE at 37°C.
4. Passaging of mesenchymal stromal/stem cells.
  a. Culture MSCs on standard cell culture plasticware. When the monolayer is at least 80 % confluent, passage them but not sparser than 1:5.
  b. Change media twice per week.

***Note:*** If possible, always plan your experiments with low passage cells (P≤9), which have been in culture for at least one passage. Primary bone marrow derived MSCs enter senescence program above passage 10 and stop growing. We also recommend confirming the mycoplasma free status of cultured cells before using them for an experiment.

5. Thawing MSCs.
  a. Thaw the cryovial in your hands, avoid water bath (contamination risk higher).
  b. Pipette 1 ml cold growth media dropwise on the cells and transfer into 8 ml of cold medium in a 50 ml falcon tube. Spin down for 5 min at 300 g.
  c. Aspirate the medium and resuspend in warm medium, transfer into a T-75 and place at 37°C.
6. Freezing MSCs.
  ***Note:*** A T-175 fully confluent with MSCs contains approximately 5 million cells (this is quite variable) and can be divided into 6 cryovials.
  a. Wash the cells with 15 ml of PBS.
  b. Aspirate the PBS and use 3 ml of TrypLE Express to detach cells, incubate at 37°C until the monolayer is loose.
    ***Note:*** This happens usually within maximum of 3 min, but it can vary per MSC donor and confluency. To avoid excess trypsinization resulting in cell clumps and lysis observe the cells under the microscope after adding TrypLE Express.
  c. Use 3 ml of media to collect the cells from the bottle into a 15 ml tube.
    ***Note:*** In the rare case where some cells remain attached after this step, use another 15 mL of PBS to wash the remaining cells off the flask bottom and/or repeat the TrypLE Express treatment.
  d. Spin down for 5 min at 300 g.
  e. Aspirate the media and carefully resuspend the cell pellet in 3 ml of growth media.
  f. Add 3 ml of 2x MSC freezing solution dropwise into the cell suspension and turn the tube upside down a couple of times to mix the solution.
  g. Divide into 6 cryotubes, 1 ml per tube.

### Rescue of busulfan-treated organoids with MSCs

**Timing: 9–14 days**

**Figure 2:**
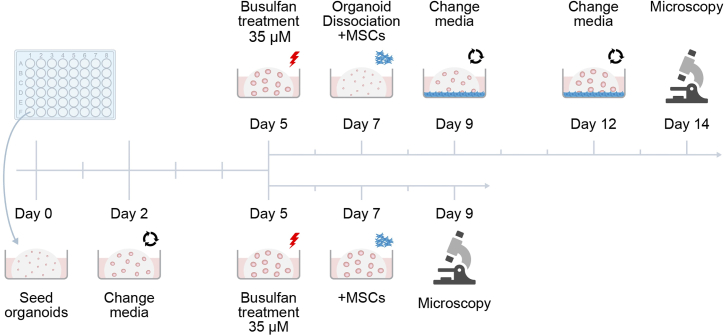
Timeline of the co-culture of duodenal organoids with MSCs Single cells are seeded and grown into organoids. The organoids are exposed to an insult on day 5, which is removed 2 days later and MSCs are added. In the long protocol, the organoids are consequently dissociated into single cells before the addition of MSCs. On day 9, the short experiment is finished, while the organoids in the long experiments are allowed to grow into day 14. At the end of both experiments, the droplets are imaged with a light microscope.

These instructions explain how to perform two different intestinal epithelium injury rescue assays with MSCs (Figure 2). The 9-day Short Protocol tests the ability of organoids to withstand and recover from a chemical insult. The 14-day Long protocol probes the longer-term effects inflicted on the cells within the organoids and their ability to grow organoids from single cells after insult. The instructions apply for both protocols unless specifically mentioned in the title or text.

**CRITICAL:** Always keep matrigel on ice to prevent unwanted polymerization.

**CRITICAL:** Since the quality of conditioned media can vary, ensure that the RCM, NCM and WCM used in EM +/+/+ during the whole experiment all come from the same batches. Since the assay is very sensitive, changing conditioned media batches during experiment will likely result in increased variability. CM batches can also be combined to limit batch-to-batch variation, which can be assessed by comparing organoid growth.

***Note:*** For practical reasons, it is advisable to start the Short assay on a Wednesday, which will make it end on the Friday of the following week.

7. Preparations.
  a. Prepare cold AdDF+++ and warm EM +/+/+.
  b. Keep matrigel diluted 50 % with EM +/+/+ on ice.
8. Plating the organoids (Day 0).
  a. Gather 3 wells with fully grown organoids from a 24-well plate with 1 ml of cold AdDF+++ and transfer them to 15 ml tube with 12 ml cold AdDF+++.
  b. Centrifuge at 200 g for 5 min at +4°C.
  c. Aspirate the medium from top and carefully add 1 ml TrypLE on top without disturbing the pellet.
  d. Incubate for 5 min in 37°C waterbath.
  e. Pipette up/down a few times to break the pellet and incubate for 2–5 min in 37°C. Use a microscope to confirm most of the solution consists of single cells (Figure 1).
  f. Add 10 ml of cold media to dilute TrypLE and spin down for 5 min at 200 g at +4°C.
  g. Resuspend the pellet in 300 μl EM +/+/+ and count the cells.
  h. Combine matrigel with EM +/+/+ and cells in a manner resulting in 500 cells per 10 μl droplet consisting of 2/3 matrigel and 1/3 EM +/+/+ and cells.
    ***Note:*** Resuspend the matrigel+EM+cells mixture well by gently pipetting up/down with a p200 several times before pipetting the droplets to ensure homogeneous distribution of cells among droplets. Overseeding may complicate data analysis, see limitations and problem 3.
  i. Immediately after resuspension pipette a droplet per well of a 48-well plate and place it in a 37°C incubator for 15 minutes to solidify the droplets.
  j. Add warm 250 μl of EM +/+/+ with 10 μM ROCK inhibitor carefully on top and return the plate to the incubator for 2 days.
9. Changing media (Day 2).
  a. Prepare warm EM +/+/+.
  b. Confirm the organoids are growing as expected i.e., the single cells have started to form little organoids.
  c. Aspirate the media from wells and add 250 μl of EM +/+/+ on the wells (without ROCK inhibitors from now on).
  d. Incubate the plate for 2 days at 37°C.
10. Busulfan treatment (Day 5).
  **CRITICAL:** Busulfan is toxic and carcinogenic, handle with care and consult manufacturer's Safety Data Sheet for details.
  a. Prepare warm EM +/+/+.
  b. Aspirate the media from wells and add 250 μl of EM +/+/+ with 35 μM busulfan to the wells.
    ***Note:*** The concentration of busulfan was chosen empirically as the most optimal to induce enough damage but not kill the intestinal organoids and to have a good window for capturing the rescue effects of MSCs. We have tested the concentrations of busulfan ranging from 0.35–35 μM. The stock solution concentration used was 6 mg/ml (24.4 mM) and it was stored at +4°C. The stock solution contains dimethylacetamide and macrogol 400.
  c. Incubate the plate for 2 days at 37°C.
11. Dissociation into single cells (Long protocol) and the addition of MSCs (Short and Long Protocol) (Day 7).
  a. Prepare warm MOCC media by combining 6/7 of EM +/+/+ with 1/7 of MSC medium.
    ***Note:*** This ratio was chosen empirically as the most suitable for both organoids and MSCs from the tested range of 1:1– 1:6.
  b. Take a full T-175 with MSCs and aspirate the media. Add 3 ml of TrypLE and incubate at 37°C until the cells detach.
  c. Add 10 ml of MSC medium into the flask and pipette gently up/down to gather any cells remaining attached to the bottom. Transfer the cells to a 15 ml tube.
  d. Spin down for 5 min at 300 g.
  e. Remove supernatant, resuspend in 2 ml of MOCC medium and count the cells.
    ***Note:*** MSCs consist of a heterogeneous population and are notoriously difficult to count accurately with standard automated cell culture counters utilizing Trypan Blue staining. It is advisable to take increased counts, such as 4–6 instead of the usual 2 to get statistically relevant results (also makes it easier to discard outliers). MSCs are most accurately counted either manually or with devices such as CASY Counter.
  f. Depending how many wells are needed in the experiment, combine an appropriate amount of cells with MOCC medium resulting in 40k MSCs/ml.
  g. Aspirate medium from the wells of the organoid plate.
  h. Mix the tube with MSCs well and add 250 μl of medium and cell mix per well (=10k MSCs).
  i. Incubate the plate for 2 days at 37°C.
    ***Note:*** Long protocol only: Perform steps 11a-11f as above. Dissociate busulfan treated organoids into single cells and pipette into droplets according to steps 8a-8i from Day 0. After letting the droplets solidify for 15 min, proceed according to steps 11h-11i above.
12. End of experiment (Short protocol)/Changing media (Long protocol) (Day 9).

Short protocol: Proceed to section microscopy and data analysis

Long protocol: Change media similar to Day 2 except with MOCC medium.

13. Changing media (Long protocol) (Day 12).

Change the media on the cells like day 9.

14. End of experiment (Long protocol) (Day 14).

Proceed to section microscopy and data analysis.

### Microscopy and data analysis

**Timing: ∼2 h**

This section entails the acquisition of light microscope images of the organoids and their analysis with OrganoSeg and OrganoAna.

15. Preparations.
  a. Download and install OrganoSeg from https://github.com/JanesLab/OrganoSeg.
  b. Download the data analysis Excel tool OrganoAna from https://github.com/B-P-R-C/Lorenowicz/blob/main/OrganoAna%20-%20ExpectedResults.xlsm.
16. Image acquisition.
  a. Place the organoid plate under a microscope and select 1.25x objective.
  b. Adjust focus level until most organoids are in focus and take bright-field images of each droplet.
  c. Include a scalebar in the image so that you can correlate pixels to micrometers in OrganoSeg.
    ***Note:*** This needs to be done only once per objective.
  d. Open the image with the scalebar in e.g., Fiji and measure the scale. For example when using EVOS M5000 microscope, for the 1.25x objective this is 346 pixels/2000 μm and for the 4x objective the scale is 358 pixels/550 μm.
17. Organoid segmentation and extraction of metrics with OrganoSeg.
  a. Launch OrganoSeg (Figure 3).

**Figure 3:**
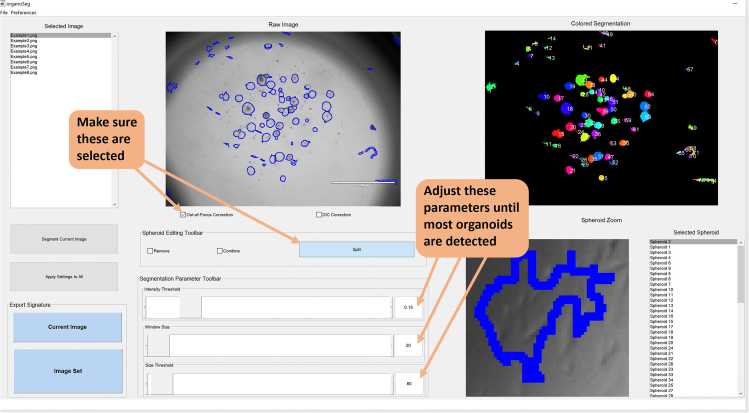
Overview of important parameters in OrganoSeg Good starting values for Intensity threshold, Window size and Size threshold are 0.2, 20 and 80, respectively.
  b. Go to File -> Open -> New Image Set and select a maximum of 72 images for analysis and click “Open”.
    ***Note:*** Ensure the order of the files loaded is known as the filenames will not be present in the OrganoSeg output file, only their order.
  c. Check the “Out-of-Focus Correction” checkbox.
  d. In the Spheroid Editing Toolbar, click “Split”.
  e. Go to Preferences -> Calibration and type how many pixels correspond to how many micrometers.
  f. Go to Preferences -> Metrics and check all the checkboxes for different parameters (from Area to Homogeneity).
    ***Note:*** This will ensure that the data columns in the OrganoSeg output file are always in the same order. Not doing this will result in failed or erroneous analysis, because OrganoAna reading the output file assumes the columns are in a certain position.
  g. Select the first image from the list on the left and set “Intensity Threshold” to 0.2, “Window Size” to 20 and “Size Threshold” to 80. We have empirically concluded these to be a good starting point but exact settings will depend on the culture and images taken. Adjust the values until all or most organoids are properly detected. If an organoid is incorrectly split into several parts, use the combine tool to segment these into one organoid.
    ***Note:*** At this point it does not matter if excessive background objects are detected as well, these will be filtered from the data downstream based on their non-organoid geometry (such as lack of roundness). However, the total number of objects detected per image should not exceed 2000 (see limitations).
  h. Click “Apply Settings to All” to segment all images.
  i. Browse the images to confirm the settings work for all images. Adjust the settings on individual images if necessary.
  j. To save the segmented images, select File -> Save -> Image Set.
  k. Export the data by clicking “Image Set” in the “Export Signature” box in the lower left corner. Name the file and save as an Excel file.
18. Data quantitation and visualization with OrganoAna.
  a. Open the OrganoSeg output file and OrganoAna (Figure 4).

**Figure 4:**
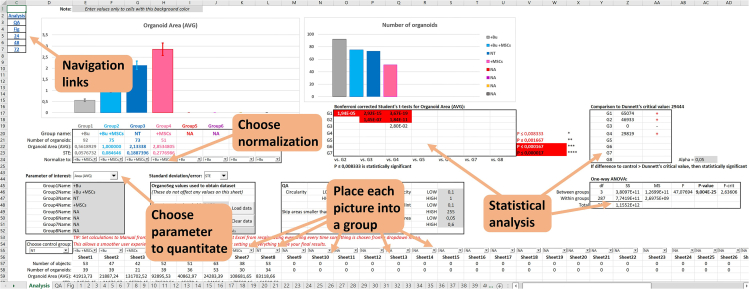
Overview of important parameters in OrganoAna Type the name of the export file from OrganoSeg into the Input file name and click Load data. Place the data replicates into groups and select the group to normalize to. For easy export into graphics editor programs, a black & white skeleton of the graph without any markings is available on the Fig sheet.
  b. Enter the parameters used in steps 9 and 11 to “OrganoSeg values used to obtain dataset”. These are Intensity Threshold, Window Size, Size Threshold and Scale. This is only for record keeping and they are not needed for the analysis.
  c. Type the name of the output file into “Input file name”.
  d. Click “Load Data” button. This will transfer the values from the OrganoSeg output file to OrganoAna sheets 1–72. Clearing and loading data takes about 8 minutes on a desktop PC with 8 CPU cores at 4 GHz and Excel will be unresponsive during this time.
    ***Note:*** If clicking the button immediately causes “Run time error ‘9’: Subscript out of range”, ensure the typed file name is correct and both OrganoAna and the OrganoSeg output file are simultaneously open in Excel. If the OrganoSeg output file contains less than 72 sheets (i.e. images) as is most often the case, there will be the same error in the end. In this case, just click “End” button on the error window, all the data was nonetheless successfully copied.
  e. The names for up to 8 different conditions can be set on the left (Group1name-Group8name).
  f. Place each image dataset into its corresponding condition using the dropdown menus.
    ***Note:*** Since OrganoAna contains a lot of data and links, the user experience may become slow at times. For example, there may be a delay of several seconds every time something is selected from a dropdown menu. To mitigate this, it is advisable to set Excel not to recalculate everything after every step. This can be set from Formulas -> Calculation Options -> Manual. Do not forget to turn it back on to Automatic when you want to see the results!
  g. Select your “Parameter of Interest” from the dropdown menu, average area is the default.
  h. In order to easily compare different conditions, select the group used for normalization at the bar graph.
    ***Note:*** An easily exportable version of this graph without any labels can be found on sheet “Fig” for further downstream editing. This tab also contains the raw data of each group.
  i. Bonferroni corrected Student’s t-tests, One-way ANOVA and Dunnett’s post-hoc analysis for statistical significance between different conditions can be found on the right.
    ***Note:*** Additional settings can be found in the QA box. By default, only Size, Circularity and Perimeter vs. Area ratio are used to filter out background objects, which are not round. A breakdown of objects in each dataset can be found in the QA tab.

## Expected outcomes

**Figure 5:**
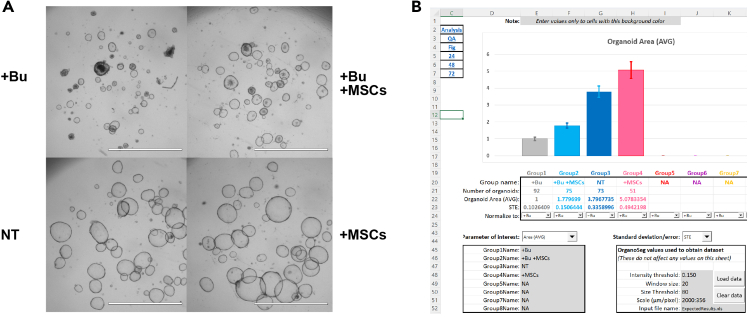
Example images of the day 9 organoids grown according to the short protocol Scale bar is 2000 μm (A). Quantitation of the images in (A) using OrganoAna (B). Data are represented as mean ± SEM. Bu = busulfan, MSCs = mesenchymal stromal/stem cells, NT = non-treated.

The protocol described here provides a relatively straightforward method to investigate interactions between intestinal epithelium and mesenchymal stromal/stem cells. In our study, the focus was on how MSCs can promote the recovery of intestinal epithelium following chemotherapy-induced damage. Figure 5 shows an example of a rescue experiment performed according to the Short protocol. These data demonstrate that MSCs increased the size of intestinal organoids treated with busulfan by approximately 1.9-fold. The MSC-mediated rescue of busulfan-induced damage typically ranges from 1.5- to 2-fold, depending on the MSC donor.

## Quantification and statistical analysis

The numbers provided by OrganoSeg are read by OrganoAna and then each detected object passes through a number of checkpoints designed to filter background noise and other non-organoid objects detected by the OrganoSeg algorithm. Minimizing the background with the settings usually also means leaving organoids undetected. Thus, it is better to err on the side of detecting too many objects, because the non-organoid objects can be filtered out. This takes place using the following parameters (the default values have been acquired experimentally).

1. Circularity of the object is calculated by dividing two values provided by OrganoSeg, MajorAxisLength with MinorAxisLength. This results in values greater than 1 and with the default settings, values below 0.5 and greater than 1.6 are filtered out.
2. Eccentricity is given by OrganoSeg but not currently used for filtering. The default accepted values are 0.1–1 i.e., everything.
3. MeanPixelInt is similarly not used. These values are the lower and upper limit of the pixel intensities for objects that are included in the dataset and since 8-bit image data has values from 0 to 255, the default values 0.1–255 are meant to not filter out anything.
4. Perimeter/A ratio is something that decreases as organoid size increases. This parameter is useful for detecting and filtering longitudal, thin non-spherical objects typically found in the background. The default accepted range is 0.05–0.6 i.e., the filtering happens on the “upper end” of the scale where such objects reside.
5. Finally, the size is used to exclude miniscule objects, which are typically optical aberrations or false positives of the detection algorithm. We have decided to exclude objects smaller than 300 μm^2^.

Failure to pass any of these tests will result in the exclusion of that object from the dataset.

The Student’s t-tests between different data groups are calculated with two-tailed distribution and two-sample unequal variance (Excel function TTEST(Array1;Array2;2;3). Subsequently, the P-values are corrected with the Bonferroni method. In Dunnett’s test, we used two-tailed comparison assuming unequal group sizes.

## Limitations

### The model

This co-culture model is a simplified representation of the *in vivo* situation. It is its strength and limitation at the same time. With the current set up one can investigate the interaction between MSCs and the intestinal epithelium. However, the regenerative capacities of MSCs *in vivo* can be influenced by multiple factors such as the presence of inflammatory cytokines or immune cells. To address the contribution of these factors to the MSC regenerative effects the current model would need to be modified, resulting in increased complexity. This would probably also influence the reproducibility of the data.

Perhaps the most challenging factor of such a new experimental setup would be to find media, which is compatible with all cell types present. We do not know how exactly the MOCC media influences both the organoids and MSCs.

In this co-culture method there is no stringent separation of organoids and MSCs, thus one cannot distinguish whether the beneficial effects are exerted by MSCs via cell-cell interactions or paracrine signaling. This can be addressed by physically separating the two cultures, for example by using Transwell inserts.^1^

When using a chemical treatment of the cells such as busulfan in our case, it is important to titrate it, in order to determine the concentration range where the assay works quantitatively. If the concentration is too low, it might be difficult to distinguish the effect from positive control whereas too high concentration will result in no surviving organoids. For the chemicals used in clinics, like busulfan, doses administered in the patients might be used as a starting point. However, it needs to be taken into consideration, that *in vitro* the effect of such a chemical is more direct than *in vivo*. Thus, the concentration may need to be adjusted to the needs of the assay.

The organoids in this protocol consist mostly of stem-like cells, which do not faithfully copy the composition of the intestinal tissue *in vivo*. As we have not differentiated the organoids in this model, the contributions of different cell types within the duodenum are not taken into consideration.

### Data analysis

Overseeding the matrigel droplets with cells will result in more overlapping organoids, which may decrease the quality of the image analysis. In general, the number of cells per matrigel and the end date of the experiment can be modified to yield a density most optimal for segmenting the organoids.

OrganoAna is a simple Excel file and therefore comes with certain technical limitations.

First, OrganoAna is currently limited to analyzing 72 images simultaneously. This is due to the number of sheets inside the Excel file as each image data is saved on its own sheet. We chose to include 72 sheets for the data, because this allows the analysis of a full 24-well plate with 3 matrigel droplets per well.

Second, the number of objects per sheet that OrganoAna reads from the OrganoSeg output file is 2000. In theory, each analyzed image contains at least 500 objects, because we seed 500 cells. However, in practice the number of objects detected is much greater, because all images contain background objects/shadows/artefacts, which will be detected as well. At best, we have observed a bit more than 1000 objects per image. In order to minimize the possibility of excluding organoids from the analysis, we chose 2000 as the maximum number of objects as it is about double what we have seen in practice. Thus, the probability of missing an organoid is exceedingly low. If there is a lot of clutter on the screen, confirm the number of objects by scrolling down the “Selected Spheroid” list on the lower right side of the screen in OrganoSeg. These 2000 objects are then processed and non-organoid looking geometry is filtered out according to the settings in the QA-box of in OrganoAna. Of the remaining objects, a maximum of 500 objects per sheet are transferred to “Analysis” sheet for calculations. Finally, the data can be grouped into a maximum of 8 conditions.

It can be argued that imaging only one X-Y plane of the organoid droplet leaves some organoids out and introduces a source of inaccuracy to the experiment. However, a mitigating factor to this is the fact that the seeded cells still tend to sink down after pipetting the droplet and before it has solidified enough and accordingly, most of the organoids are indeed found in the bottom of the droplet. Also to mitigate this, it is important to keep the droplet size small enough, thus 10 μl was deemed suitable for the assay.

## Troubleshooting

### Problem 1

The pellet of organoids does not dissolve into single cells or the solution becomes slimy, day 0, step 8e.

### Potential solution

The incubation time with TrypLE will vary per experiment and is mainly dependent on the number of cells. If the solution appears slimy (likely DNA), cell lysis may have occurred and the incubation time and/or pipetting should be decreased. If clumps remain after pipetting up/down, incubating a short while after pipetting may result in more single cells. The addition of ROCK inhibitor into the cell dissociation reaction may yield more live cells.

### Problem 2

Droplets contain different amounts of organoids, step 8h.

### Potential solution

Pipet the organoid fragment-matrigel mixture more thoroughly before plating. The viscous nature of matrigel makes it more difficult to create a homogeneous mixture.

### Problem 3

Image analysis: OrganoSeg is unable to properly segment a droplet of overlapping organoids due to overseeding despite using the Split function, step 17g.

### Potential solution

If this happens only with a few organoids, it will not significantly affect the data and this can be statistically compensated for by adding more wells i.e., making the dataset more robust. If the overlap is persistent and the segmentation is poor, consider analyzing the data at an earlier time point when the organoids are smaller and therefore do not overlap. It might even help to analyze them at a later time point when they are bigger and thus less likely completely inside each other allowing better detection by the Split function in OrganoSeg. However, probably the easiest way to solve this issue is to decrease the number of seeded cells in the beginning. In general, the seeding of 500 cells gave the best results in and that number should be a fair starting point also if other types of organoids are used.

### Problem 4

Data analysis: OrganoAna is excluding my data!

### Potential solution

This only happens if the .xls output file from OrganoSeg consists of more than 72 tabs. Only load and analyze up to 72 images in OrganoSeg at one time in step 17b to accommodate this OrganoAna shortcoming.

### Problem 5

Data analysis: The name of the input.xls file is entered into OrganoAna but the data is not transferred to OrganoAna sheet in step 18d.

### Potential solution

Ensure you have both files open in Excel simultaneously: OrganoAna and the input file.

## Resource availability

### Lead contact

Further information and requests for resources and reagents should be directed to and will be fulfilled by the lead contact, Magdalena Joanna Lorenowicz (lorenowicz@bprc.nl).

### Technical contact

Technical questions on executing this protocol should be directed to and will be answered by the technical contact, Kari-Pekka Skarp (skarp@bprc.nl).

### Materials availability

This study did not generate new unique assets.

### Data and code availability

The OrganoAna Excel tool generated during this study is available at https://github.com/B-P-R-C/Lorenowicz/blob/main/OrganoAna%20-%20ExpectedResults.xlsm and is archived at Zenodo: DOI https://doi.org/10.5281/zenodo.17602501.

## Acknowledgments

We thank Prof. Calvin Kuo for kindly providing HEK293T-HA-Rspo1-Fc cells and Prof. Hans Clevers for kindly providing L-cells stably expressing Wnt3A and HEK293 cells stably expressing Noggin-Fc. This research was financially supported by Kika (project 363), an Alexandre Suerman Stipend of the UMC Utrecht and KF Hein Fonds.

## Author contributions

B.Y.-A. developed and validated the protocol with input from S.A.J., C.A.L., and M.J.L. K.-P.S. contributed to the data analysis, wrote the manuscript, and prepared figures, and B.Y.-A. and M.J.L. provided input on manuscript preparation. M.J.L., B.Y.-A., and C.A.L. were involved in proof reading, and all authors read and approved the final version of the manuscript.

## Declaration of interests

The authors declare no competing interests.
